# Prevalence and predictors of chronic disease among rural and medically underserved populations using smokeless tobacco

**DOI:** 10.3389/fpubh.2025.1565910

**Published:** 2025-05-09

**Authors:** Elaine Sang, Susan Silva, Sarah Grenon, Courtney Swinkels, Shamatree Shakya, Salsa Deshmukh, Laura J. Fish, Mariana Da Costa, Leigh Ann Simmons, Herbert H. Severson, Kathryn I. Pollak, Devon Noonan

**Affiliations:** ^1^New Courtland Center for Transitions and Health, School of Nursing, University of Pennsylvania, Philadelphia, PA, United States; ^2^Leonard Davis Institute of Health Economics, University of Pennsylvania, Philadelphia, PA, United States; ^3^School of Nursing, Duke University, Durham, NC, United States; ^4^School of Nursing, University of North Carolina Wilmington, Wilmington, NC, United States; ^5^Duke Cancer Institute, School of Medicine, Duke University, Durham, NC, United States; ^6^Department of Family Medicine and Community Health, Duke University, Durham, NC, United States; ^7^College of Health and Human Sciences, School of Nursing, Western Carolina University, Cullowhee, NC, United States; ^8^Betty Irene Moore School of Nursing, University of California, Davis, Sacramento, CA, United States; ^9^Oregon Research Institute, Springfield, OR, United States; ^10^Department of Population Health Sciences, School of Medicine, Duke University, Durham, NC, United States

**Keywords:** chronic disease, rural, smokeless tobacco (SLT), medically underserved area (MUA), cessation interventions, nicotine dependence

## Abstract

**Introduction:**

Smokeless tobacco (SLT) is significant public health problem in the U.S. and is associated with chronic diseases, which includes both physical and mental health conditions. Inequities in use exist as rural and other medically underserved populations use SLT more than that of the general population. Our study examined prevalence of chronic disease and identified associated risk factors among rural and medically underserved populations using smokeless tobacco.

**Methods:**

We conducted a cross-sectional analysis of baseline data (*N* = 532) from a clinical trial promoting SLT cessation among adults living in rural and/or medically underserved areas and examined the rates of eight chronic diseases: cancer, lung disease, heart disease, stroke, mental health conditions, diabetes, arthritis/orthopedic conditions, and hypertension. Multivariable logistic regression was used to identify risk factors for four of the most common chronic diseases among study participants.

**Results:**

Over 60% of our sample of rural and medically underserved adults who use SLT also have at least one chronic disease. The most common chronic diseases were hypertension (38%), arthritis/orthopedic (23%), mental health (21%), and diabetes (12%). Increasing age and poor/fair health were associated with having hypertension, arthritis/orthopedic conditions, and/or diabetes. In addition, drinking alcohol <5 days per week was associated with having diabetes. Meanwhile, greater nicotine dependence, marital status, and having stained teeth were associated with having a mental health condition.

**Conclusions:**

Findings may inform the development of SLT cessation interventions as part of broader chronic disease management programs and as part of secondary prevention to minimize tobacco related morbidity.

## 1 Introduction

Smokeless tobacco (SLT), encompassing products such as chew, snuff, and dip, is often perceived as a less harmful alternative to cigarette smoking (1–3). However, it remains a major public health concern with over 300 million people using SLT worldwide (4, 5) and more than 650,000 deaths linked to it each year (5). SLT use has been linked to substantial global mortality and morbidity (4, 5) and is associated with a wide range of chronic diseases, including hypertension (6), cardiovascular disease (6–9), cancer (4, 10, 11), stroke (8, 9, 12), and mental health conditions (13, 14). Interestingly, although little literature is available, SLT use has not been associated with arthritis and other chronic inflammatory diseases (15). These health effects exacerbate the overall health burden, especially for those already at higher risk of these diseases, making it more difficult to address health challenges at both the personal and public health levels.

Within the United States (U.S.), ~5.2 million adults use SLT, most of them being non-Hispanic white men (16). SLT use is notably more common within rural and underserved communities compared to within urban areas, with rates of current use being two to three times higher in rural areas compared to their urban counterparts (16–21). In these communities, SLT use is deeply rooted in cultural norms, contributing to its widespread acceptability (20, 21). Rural and underserved communities face compounded health risks when tobacco use coexists with chronic diseases, and limited healthcare resources and significant health disparities delay diagnoses and worsen health outcomes. Compared to urban areas, rural and underserved areas are disproportionately affected by chronic disease burdens and have fewer resources to address them (22–25). Furthermore, people in these areas tend to experience higher rates of disability and mortality and have lower health literacy than their urban counterparts (25–31). All of this underscores the urgency of targeted interventions that address both SLT cessation and chronic disease prevention in these populations.

While studies, reviews, and meta-analyses have examined whether SLT use is associated with chronic diseases (4, 7, 9, 10, 14) and assessed the effectiveness and feasibility of cessation interventions (18, 19, 32, 33), little is known about the relationship between chronic disease and associated risk factors among those using SLT and residing in rural or medically underserved communities. Addressing this gap could help inform the development of interventions and public health strategies specifically tailored to such populations, aiming to reduce their unique health challenges and disparities. Therefore, our study focused on examining the prevalence of chronic disease and associated risk factors among those using SLT and residing in rural or medically underserved communities.

## 2 Methods

### 2.1 Overview

This study is a cross-sectional secondary analysis of baseline participant data from a parent randomized control trial (RCT), which compared the efficacy of a text-based intervention (#EnufSnuff.TXT) to an established evidence-based program in reducing SLT use among adults living in rural and/or medically underserved communities (18). In brief, the original study recruited, via social media, 532 people who used SLT and lived in a rural or medically underserved area of the country (18). Most participants resided in North Carolina (36%), South Carolina (10%), West Virginia (9%), Texas (8%), Mississippi (8%), Kentucky (7%), and Pennsylvania (7%) (18). The parent RCT was approved by the Duke University Institutional Review Board (18).

### 2.2 Participant eligibility and recruitment

Participants eligible for the parent RCT met the following inclusion criteria: (1) at least 18 years old, (2) have a cell phone with unlimited texting service, (3) reside in a rural area and/or medically underserved area, defined as areas with a shortage of primary care health services [Rural-Urban Commuting Codes of 4–10 (34) and Health Resources and Services Administration Medical Underservice Index of 62 or lower (35)], (4) willing to participate in a SLT cessation program, and (5) used SLT for at least three times per day for the past 30 days. Those using additional tobacco products, such as cigarettes, were not excluded unless they were unwilling to abstain from those products while participating in the RCT. Those interested in participating were directed to complete a contact form, where they provided their contact information. Research staff sent electronic screeners to those with valid emails to assess eligibility criteria and consented those who were deemed eligible.

### 2.3 Baseline survey

After providing informed consent, participants completed a baseline survey, which collected sociodemographic characteristics, clinical characteristics, SLT use, oral health, and chronic diseases.

#### 2.3.1 Sociodemographic characteristics

Sociodemographic characteristics include age, gender, race, marital status (single, divorced, widowed, married, or living with partner), highest educational attainment (did not complete high school, high school diploma, GED, vocational training, formal education after high school), and health insurance (none, private health insurance, public health insurance such as Medicare and Medicaid).

#### 2.3.2 Clinical characteristics

Clinical characteristics include current alcohol use, depression symptoms, and perceived health status. Current alcohol use was assessed via the following question: “How many days per week did you have at least one of any alcoholic beverages (during the past 30 days)?” Depression symptoms were assessed via the Patient Health Questionnaire-2 (PHQ-2) (36). This questionnaire contained two questions, each of which was on a zero-to-three-point scale with zero being not at all and three being nearly every day (36). The total PHQ-2 score (possible range: zero to six) was derived by summing the scores from the two items (36). A total score of three to six indicates a positive depression screening while a total score lower than three indicates a negative depression screening (36). For perceived health status, participants rated their general health as either excellent, very good, good, fair, or poor (37, 38).

#### 2.3.3 Smokeless tobacco (SLT) and other tobacco use

Participants were asked how old they were when they started using SLT regularly, the number of cans and/or pouches of SLT they used per typical week, and the number of chews and/or dips they used per typical day. They were also asked if they have used other tobacco products, including cigarettes and e-cigarettes. Participants were administered the six-itemed Fagerstrom Test for Nicotine Dependence-Smokeless Tobacco (FTND-ST) to assess current severity of their nicotine dependence (39). The nicotine dependence total score was derived by summing the six items, with a possible total score range of 0–10 and higher score indicating greater dependence (39).

#### 2.3.4 Oral health

The baseline survey inquired whether participants had any of the following oral health problems: (1) bleeding gums, (2) receding gums, (3) stained teeth, (4) mouth scores, and (5) bad breath. Answer choices include “no” and “yes”.

#### 2.3.5 Chronic diseases

Participants were asked whether a health care provider, such as a physician or advance practice provider, had ever told them that they had any of the following: (1) cancer, (2) lung disease, (3) heart disease, (4) stroke, (5) mental health condition (such as depression, anxiety, and schizophrenia), (6) diabetes, (7) arthritis/orthopedic condition, and (8) hypertension. Answer choices for each include “no,” “yes,” “don't know,” and “prefer not to answer.”

### 2.4 Data analysis

Data cleaning, descriptive data analyses, and inferential data analyses were performed using SAS 9.2 statistical software (40).

#### 2.4.1 Data cleaning

Marital status was categorized based on whether or not participants were married or living with their partner while educational attainment was categorized based on whether or not the participants received post-secondary education. Meanwhile, health insurance was categorized based on whether the participants had health insurance. Alcohol use was dichotomized as follows: (1) <5 days per week and (2) 5–7 days per week, and responses to perceived health status were dichotomized into (1) poor or fair health and (2) good to excellent health. The number of SLT cans and/or pouches used per week was dichotomized (1) less than three cans and/or pouches and (2) three or more cans and/or pouches. The number of years using SLT was determined by subtracting participants' age when they started using SLT regularly to the date when they completed the baseline survey. There was significant missing participant data for the oral health question inquiring if participants had bad breath, leading to its exclusion from the data analyses. Participant answer choices of “don't know” and “prefer not to answer” to questions inquiring chronic disease were treated as missing data.

#### 2.4.2 Descriptive data analyses

Means, standard deviations, and ranges were calculated for age, number of chews and/or dips per day, nicotine dependence total score, and years of SLT use. Frequencies and percentages were calculated for the other variables. In addition, the percentage of those with two or more chronic diseases and the median, range, and interquartile range of chronic disease per participant was calculated.

#### 2.4.3 Inferential data analyses

Our participant sample size provided at least 80% statistical power for bivariate and multivariable logistics regression modeling, assuming a small effects size (ORs ranging from 1.50 to 1.59), a two-tailed significance set at an alpha level of 0.05 per test, and having up to seven predictors in each multivariable model. The four most common chronic diseases as identified from the descriptive data analyses were included in subsequent inferential data analyses. Bivariate and multivariable logistic regression analyses were done to identify predictors of each of the four chronic diseases. Gender, race, and health insurance were omitted from these regression analyses due to lack of variability.

Bivariate analyses were conducted to identify associations between each sample characteristic and chronic disease outcome. For each of the four most common chronic diseases, we retained only those sample characteristics significantly related to the chronic disease for the initial multivariable logistic regression model. A backward elimination variable selection approach with a stay criterion of 0.05 was applied to the initial multivariable logistic regression model for each chronic disease outcome. Although positive depression screen was a significant predictor for mental health conditions in the bivariate analysis, depression screening results were omitted from the final multivariable regression model for mental health conditions as depression is considered a mental health condition. This approach was used to reduce the initial model to a final parsimonious model that included only statistically significant predictors of each chronic disease examined. Effect sizes of the predictors in the final, parsimonious model for each chronic disease outcome were estimated with adjusted odds ratios (aORs) and their 95% confidence intervals (CIs). Non-directional statistical tests were conducted with significance set at an alpha level of 0.05 per test.

## 3 Results

### 3.1 Sample characteristics

The parent RCT recruited 532 participants, all of whom participated in the baseline survey and are thus in the secondary data analysis. The mean age was 44.9 years (range: 19–79), with 33% of the sample older than age 50 years. The sample was primarily males (99%) and white (97%). Most were married or living with a partner (83%), receiving post-secondary education (84%), and had health insurance (99%). At baseline, 8.2% (total: *n* = 44, with 22 within the treatment arm) of the participants reported dual use of smoking cigarettes and SLT in the past 30 days, and 4.1% (total: *n* = 22, with 11 within the treatment arm) reported dual use of e-cigarettes and SLT within the same period.

Of the participants, 26% reported consuming alcohol 5–7 days per typical week, 13% had a PHQ-2 positive depression screen, and 14% reported perceiving having fair or poor health. Around 78% of the participants reported using three to five cans and/or pouches per week, and the mean number of dips and/or chews per day was 10.2 (range: 2–30). The mean nicotine dependence total score was 5.4 (range: 0–10), while the mean years of SLT use was 26.7 years (range: <1–65 years). The most common oral health problems were stained teeth (70%) and receding gums (67%).

Sixty percentage of the participants had at least one of the eight chronic diseases listed in the baseline survey, the most common being hypertension (38%), arthritis/orthopedic conditions (23%), mental health conditions (21%), and diabetes (12%), 29.2% have two or more chronic diseases and the median number of chronic diseases was 1.0 (Q1 = 0.0, Q3 = 2.0, range: 0–5). Table 1 below provides more detail on the characteristics of the 532 participants, including the chronic diseases they reported.

**Table 1:** Sample characteristics (*N* = data available for analysis).

**Sociodemographic characteristics**	***N***	***n* (%)**	**Mean ±SD**
Age, in years	532		44.9 ± 11.9
Male gender	532	528 (99%)	
White race	528	512 (97%)	
Married/living with partner	530	442 (83%)	
Post-secondary education	531	447 (84%)	
Health insurance	495	492 (99%)	
**Clinical characteristics**			
Drinks alcohol beverage 5–7 days/week	523	136 (26%)	
Positive PHQ-2 depression screen	516	66 (13%)	
Perceived fair or poor health	532	74 (14%)	
**Smokeless tobacco (SLT) use**			
Three or more cans/pouches per week	528	411 (78%)	
Chews/dips per day	526		10.2 ± 5.4
Nicotine dependence total score	380		5.4 ± 2.2
Years of SLT use	532		26.7 ± 12.5
**Oral health**			
Bleeding gums	527	98 (19%)	
Receding gums	517	348 (67%)	
Stained teeth	526	370 (70%)	
Mouth sores	530	73 (14%)	
**Chronic diseases**			**U.S. prevalence**
			**comparison**
Hypertension (HTN)	527	201 (38%)	32.8%^*^
Arthritis/orthopedic condition (Ortho)	530	124 (23%)	26.6%^*^
Mental health condition (MH)	523	110 (21%)	20.6%^*^
Diabetes (DB)	530	62 (12%)	12.1%^*^
Lung disease	531	15 (3%)	6.5%^**^
Heart disease	530	31 (6%)	5.5%^***^
Cancer	532	25 (5%)	__
Stroke	531	8 (2%)	__

^*^Crude prevalence data from Crude Prevalence data and 95% Confidence Interval for ADULTS using the Data Analysis Tools available on the CDC Chronic Disease Indicator webpage which summarizes data from most recent BRFSS surveys capturing data for each chronic condition (most recent varied from 2016 to 2022) (41). Ortho category only includes arthritis for US prevalence (41).

^**^Lung disease only includes COPD in 2021 for US prevalence. Source: Behavioral Risk Factor Surveillance System (42).

^***^Heart disease prevalence is age adjusted. Source: National Health Interview Survey (43).

U.S. prevalence comparisons for cancer and stroke were not included in Table 1. This is because national data for these conditions are typically reported as incidence than prevalence.

### 3.2 Bivariate regression analysis

Table 2 below presents the bivariate regression results for the four most chronic diseases outcomes: hypertension, arthritis/orthopedic conditions, mental health conditions, and diabetes.

**Table 2:** Bivariate logistic regression results.

**Predictors**	**HTN Wald *p***	**Ortho Wald *p***	**MH Wald *p***	**DB Wald *p***
Age, in years	**<0.0001**	**<0.0001**	0.6328	**<0.0001**
Married/living with partner	0.9407	0.3435	**0.0170**	0.7752
Post-secondary education	0.6109	0.1375	0.9301	0.4264
Drinks alcohol 5–7 days per week	0.5707	0.6308	0.4845	**0.0088**
Positive depression screen	**0.0039**	0.2832	**<0.0001**	0.5951
Fair/poor general health	**<0.0001**	**<0.0001**	0.1521	**<0.0001**
Three or more cans/pouches per week	0.9380	0.7850	0.8259	0.5853
Chews/dips per day	**0.0166**	**0.0376**	**0.0170**	0.1919
Nicotine dependence total score	**0.0050**	0.2682	**0.0070**	0.0947
Years of SLT use	**<0.0001**	**<0.0001**	0.6454	**<0.0001**
Bleeding gums	0.8001	0.9120	0.2061	0.3621
Receding gums	0.6512	0.6521	0.7527	0.2633
Stained teeth	**0.0378**	0.2250	**0.0158**	0.5424
Mouth sores	0.0633	0.7688	**0.0314**	0.1826

HTN, hypertension; ortho, arthritis/orthopedic condition; MH, mental health condition; DB, diabetes. Bold values indicate *p*-values that were less than 0.1.

Hypertension had seven predictors: age, depression screen, perceived health status, number of chew/dips per day, nicotine dependence, years of SLT use, and stained teeth.

Arthritic/orthopedic condition had four predictors: age, perceived health status, number of chew/dips per day, and years of SLT use. Mental health condition had five predictors: marital/partner status, number of chews/dips per day, nicotine dependence, stained teeth, and mouth scores. Diabetes had four predictors: age, current alcohol use, perceived health status, and years of SLT use. To summarize, candidate predictors included sociodemographic, clinical, and oral health characteristics as described in Table 1.

### 3.3 Multivariable regression analysis

The final multivariable regression models for the four chronic disease outcomes are described in Table 3. In summary, participants with a higher age or perceived their health to be poor or fair had higher odds of having hypertension, arthritic/orthopedic conditions, and/or diabetes. Meanwhile, those who drink alcohol <5 days per week have higher odds of having diabetes. Those who were not married or living with their partner, have greater nicotine dependence, or have stained teeth have a higher odd of having a mental health condition.

**Table 3:** Final pragmatic multivariable logistic regression results.

**CD: outcome**	**Final parsimonious model: predictor**	**Adjusted OR (aOR)**	**aOR 95% CI**	***p***
HTN	Increasing age, in years	1.05	1.04–1.07	<0.0001
	Fair/poor general health	3.27	1.91–5.58	<0.0001
	Good to excellent general health *(ref)*			
Ortho	Increasing age, in years	1.06	1.04−1.08	<0.0001
	Fair/poor general health	2.95	1.72–5.08	<0.0001
	Good to excellent general health *(ref)*			
MH^*^	Not married/living with partner	2.56	1.29–5.05	0.0069
	Married/living with partner *(ref)*			
	Increasing nicotine dependence total scores	1.16	1.03–1.31	0.0175
	Stained teeth	2.02	1.07–3.80	0.0294
	Non-stained teeth *(ref)*			
DB	Increasing age, in years *(descending)*	1.08	1.05–1.11	<0.0001
	Drinks alcohol <5 days/week	3.17	1.35–7.41	0.0079
	Drinks alcohol 5–7 days/week (*ref*)			
	Fair/poor general health	2.63	1.36–5.11	0.0042
	Good to excellent general health *(ref)*		

HTN, hypertension; ortho, arthritis/orthopedic condition; MH, mental health condition; DB, diabetes; 95% CI, 95% confidence interval; adjusted odds ratio (aOR) effect size: small = 1.50–1.49; medium = 2.0–2.99; large = 3.0 or greater.

MH^*^ = PHQ2 depression screen scores was a predictor in the bivariate analysis but excluded from multivariable model.

## 4 Discussion

To our knowledge, few studies have investigated the prevalence and predictors of chronic disease in rural or medically underserved populations who use SLT. Given the high prevalence of both tobacco use and chronic disease in these populations (16–21), addressing SLT use in the context of chronic disease may be highly beneficial. These populations often lack sufficient healthcare resources (22–24), so targeting SLT cessation strategies could help mitigate the health burdens that they experience. Furthermore, implementing cessation programs that address barriers, such as access to healthcare, health literacy, and economic challenges (25, 29–31), is vital. As SLT use is rooted in cultural norms (20, 21), developing culturally relevant and community-driven interventions could also facilitate greater acceptance and participation in cessation efforts. Expanding this body of research is crucial for informing public health strategies that address health disparities and promote equity in tobacco control.

Prior literature has shown high prevalence of chronic disease among those who smoke cigarettes (44), but limited research has focused on SLT and chronic disease in rural and medically underserved areas. The rates of chronic disease found in our sample are lower than the national average for most chronic diseases among people smoking cigarettes (44). For example, we found that 23% of our participants self-reported orthopedic/arthritic conditions, compared to 53% among those using cigarettes within the 1999–2018 National Health and Nutrition Examination Survey (45). While 60% of those smoking cigarettes have reported two or more chronic diseases (44), we found that 29.2% of our participants using SLT have two or more chronic diseases. Interestingly, in a large study in India, there was minimal difference in chronic disease rates among those who used SLT compared to those who used cigarettes, suggesting that use of any form of tobacco may infer similar chronic disease risk (46). As all tobacco product cessation improves health (47), integrating SLT cessation within chronic disease secondary prevention interventions may improve health outcomes among rural and medically underserved populations.

The four most common chronic disease (hypertension, arthritis/orthopedic conditions, mental health conditions, and diabetes) observed in our sample of adults using SLT and living in rural and medically underserved areas had similarities and differences regarding risk factors. Although we saw numerous associations between demographic and SLT use factors in the bivariate analysis, after controlling for potential cofounders, there remained only a few significant associations. Older adults and those with poor/fair health were more likely to report experiencing chronic disease, similar to findings in the general population (37). Interestingly, drinking alcohol <5 days a week is a predictor for diabetes. Although this may conflict with other studies showing that less alcohol intake is protective against diabetes (48–50), our study did not account for the amount of alcohol. Furthermore, a meta-analysis suggested that the effect of alcohol intake on reducing diabetes may be overestimated (51). Thus, additional studies are needed to investigate the relationship between alcohol and diabetes among rural and medically underserved populations that use SLT.

Another interesting finding is that for three of these chronic diseases (hypertension, arthritis/orthopedic conditions, and diabetes), no SLT-specific factors (i.e., years of use or amount used) were associated with chronic disease in the adjusted models. Conversely, those who reported greater nicotine dependence and/or had poorer oral health [stained teeth, which is common in smokeless tobacco use (52)] were more likely to have a mental health condition in the adjusted model. While our current study examines a unidirectional relationship between nicotine dependence and mental health conditions, existing literature suggests that the relationship may be bidirectional (13, 14, 53). This suggests that those with mental health conditions may use nicotine products, such as SLT, as a behavioral coping mechanism (13, 14, 53). Such findings highlight the potential effectiveness of a multi-prong approach including targeted cessation initiatives alongside psychotherapy and/or psychiatric medications. Future research should further investigate this bidirectional relationship to inform more comprehensive cessation initiatives among those using SLT while having mental health conditions. By focusing on predictors, our findings may inform future secondary prevention interventions to promote earlier chronic disease diagnoses and thus health outcomes among those living in rural and medically underserved areas.

Our findings suggest that secondary prevention interventions for chronic diseases should include SLT cessation, particularly for rural and medically underserved populations. This could include health messaging that highlights the link between chronic diseases and SLT use, as well as the direct connections between disease management and SLT use (e.g., elevated BP with SLT use). Focusing on predictors, such as older age, poor general health, alcohol consumption, and nicotine dependence, may help identify individuals at higher risk and facilitate earlier chronic disease diagnosis and management, ultimately improving health outcomes.

### 4.1 Limitations

A limitation of this study is its cross-sectional nature, preventing us from determining causality. Another limitation is that a large majority of the sample consists of Caucasian males. Although reflective of the general population of those using SLT (16), this limits the generalizability of our findings to other demographic subgroups. In addition, all mental health conditions were categorized together within the parent RCT, preventing this study from identifying predictors associated with specific types of mental health conditions, such as depression and post-traumatic disorder. Future studies may consider assessing the prevalence and predictors of chronic disease, including specific types of mental health conditions, in a more diverse rural and medically underserved sample of those using SLT. Finally, several chronic conditions prevalent in rural populations and among those with a history of tobacco use, such as cancer and lung disease, were reported at low rates within the study (5 and 3%, respectively). This suggests that some conditions may have been underrepresented or undiagnosed, possibly due to limited access to healthcare within in rural areas.

## 5 Conclusion

The prevalence of chronic disease, including both physical and mental health conditions, are high in individuals living in rural and medically underserved areas who use SLT, and predictors may include older age, poor general health status, drinking alcohol <5 days per week, and higher nicotine dependence. Clinicians and researchers may consider implementing SLT cessation interventions as part of secondary disease prevention among chronically ill adults living in rural and medically underserved communities.

## Data Availability

The raw data supporting the conclusions of this article will be made available by the corresponding author, upon reasonable request. Requests to access the datasets should be directed to Devon Noonan, devon.noonan@duke.edu.
